# Browsing FAIR Transformation Product Information with FAIR-TPs

**DOI:** 10.1021/acsenvironau.5c00314

**Published:** 2026-03-06

**Authors:** Bashir Mayahi, Emma H. Palm, Emma L. Schymanski

**Affiliations:** Luxembourg Centre for Systems Biomedicine (LCSB), 81872 University of Luxembourg, 6 av du Swing, L-4367 Belvaux, Luxembourg

**Keywords:** Transformation Products, FAIR (Findable, Accessible, Interoperable, Reusable), Metabolites, Reactions, Environmental Transformations, Transformation Networks

## Abstract

Considering transformation products (TPs) in environmental studies remains a huge challenge for scientists, from identification in samples via mass spectrometry to inclusion in chemical regulation. This article introduces FAIR-TPs, a Web site to browse openly available TP data collated from literature sources. The data is sourced from several community-contributed environmental data sets on the NORMAN Suspect List Exchange (NORMAN-SLE), plus a data set from ChEMBL available through PubChem, with links to templates and contact details to encourage further community contributions. The data are compiled regularly using an open source workflow, archived and versioned on Zenodo under a CC-BY license, then processed and displayed on FAIR-TPs. FAIR-TPs currently contains 11,190 reactions involving 9,435 compounds from 11 sources, covering human and environmental transformations through to high energy water treatment reactions. A graph-based representation of the transformations (compounds as nodes and reactions as edges) is stored in a Neo4j database as a Directed Graph and made publicly accessible online through a Django Web Application. Users can retrieve the shortest directed pathways between predecessors (parents) and successors (transformation products/metabolites), search by SMARTS substructures, or explore local reaction neighborhood data on individual compounds. Interactive network visualizations provide ways for users to view multistep transformations in a smooth, user-friendly interface, while exploring transformation pathways. The compound/reaction metadata provide links to further information about the chemicals and data sources. Key statistics, including number of reactions/compounds, top compounds, reaction types, and mass differences are summarized from the current data set. FAIR-TPs is designed to be a public resource to support suspect and nontarget screening workflows, helping scientists identify data gaps and interpret complex transformation reactions. The FAIR-TPs website is openly available at https://fairtps.lcsb.uni.lu.

## Graphical abstract

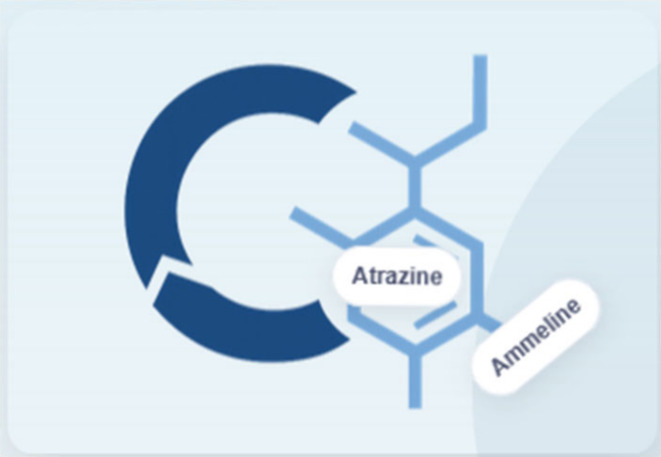

## 1 Introduction

Nontarget analysis often reveals thousands of features in environmental samples, with most of these features remaining unannotated.^1^ Transformation products (TPs) are challenging to annotate in this context, due to few reference standards being available, a scarcity of spectral data, and a lack of verifiable analytical information.^2^ Many TPs also remain either undocumented or insufficiently documented, such that their structures are not listed in structure libraries.^2^ While various methods exist to predict TPs to improve annotation success, these are inherently prone to overprediction and combinatorial explosion^2^—also due to a lack of known TP data to improve the prediction accuracy. Thus, a “chicken and egg” information gap arises during annotation. Closing this information gap to be able to both accurately annotate TPs and track their pathways back to their potential parents (or predecessors) is of the utmost importance to ensure that these complex pathways can be considered when assessing the risk posed by a given compound. TPs are also being increasingly recognized for their regulatory importance. The EU Drinking Water Directive requires member states to monitor TPs of pesticides in water intended for human consumption if there is “reason to consider that it has intrinsic properties comparable to those of the parent substance”.^3^ However, there are no TPs specified in the directive, leaving the discovery of relevant TPs, their properties, and subsequent monitoring efforts largely up to the member states. This, combined with the limited TP information available, results in very few TPs being considered during routine targeted monitoring efforts, while nontarget screening approaches remain out of reach for many routine applications.^2,4^

Suspect screening, i.e. the use of predetermined lists of chemicals suspected to be present in complex samples, has become a popular approach to improve annotation rates in nontarget screening efforts. The NORMAN Suspect List Exchange (NORMAN-SLE)^5^ has made a concerted effort to gather TP (and other) data from European and global contributors for use as suspect lists in nontarget screening, and now includes 10 lists with mapped transformations data (of a total 133 lists). Other sources of TP information includes enviPath^6^ (some of which is in the NORMAN-SLE), the Chemical Transformation Simulator reaction (CTS)^7^ library, ChEMBL (RRID:SCR_014042)^8^ and the up-coming Chemical Transformations Database (CHET) from the US EPA.^9^ While the ChEMBL and NORMAN-SLE data has been available through PubChem (RRID:SCR_004284)^10^ since ∼2020,^11,12^ with the NORMAN-SLE data updating as new entries come in, it remains difficult for most users to discover and explore this information deep down on the individual compound pages. The displayed table is also limited to single parent–TP relationships, which makes exploring the multistep nature of many reactions challenging. To facilitate NTS workflows, this data has also been integrated into the nontarget screening software patRoon^13,14^ via a compiled data set made available on Zenodo (RRID:SCR_004129),^15^ which improves workflow TP functionality, but does not provide any user browsing options.

To address these gaps, this article presents FAIR-TPs (https://fairtps.lcsb.uni.lu/), a Web site designed to facilitate the browsing of the open access TP data compiled from literature sources currently accessible in PubChem, but also encourage further contributions of open TP data to expand this knowledgebase further and contribute to the various interlinked resources and workflows. FAIR-TPs has been specifically designed to browse and explore published TP data in a Findable, Accessible, Interoperable and Reusable (FAIR) manner and potentially serve as a source of information for the development of predictive methods but is not intended as a resource to support prediction of TPs beyond these data sets.

## 2 Materials and Methods

### 2.1 Source Data

FAIR-TPs currently contains 11 data sets compiled from two primary sources (NORMAN-SLE,^5^ 10 data sets) and ChEMBL^8^ (1 data set), summarized in Table 1. Further details are given at https://fairtps.lcsb.uni.lu/stats/ and the Results section.

**Table 1:** Summary of Datasets Integrated in FAIR-TPs, Sorted by Total Entries^a^

Data set	Description, References	Total entries	Unique pairs	Unique pairs, %
S73 | METXBIODB	Reactions from BioTransformer^16,17^	2,126	1,874	88.1%
ChEMBL	TPs of drug-like bioactive molecules^8^	1,735	1,151	66.3%
S121 | EAWAGBBD	Eawag biodegradation database^6,18^	1,689	1,622	96.0%
S113|SWISSPHARMA24	Swiss pharmaceuticals and TPs^19,20^	1,603	1,305	81.4%
S68 | HSDBTPS	Metabolites from HSDB^21,22^	1,285	686	53.4%
S60 | SWISSPEST19	Swiss pesticides and TPs^23,24^	1,235	1,011	81.9%
S74 | REFTPS	Collection of literature TPs, including several PFAS reactions^25^	930	719	77.3%
S78 | SLUPESTTPS	Pesticide TPs^26,27^	294	117	39.8%
S66 | EAWAGTPS	TPs of emerging compounds^28,29^	204	89	43.6%
S81 | THSTPS	Thirdhand smoke metabolites^30^	59	56	94.9%
S79 | UACCSCEC	TPs of emerging compounds^31,32^	30	28	93.3%

a TPs = transformation products. PFAS = per and polyfluoroalkyl substances. HDSB= Hazardous Substance Data Bank. All datasets except ChEMBL are from NORMAN-SLE.

These data sets are compiled via the “Transformations” section integration of the NORMAN-SLE and ChEMBL data in PubChem (described elsewhere^11,12^ ), retrieved using open code in R available on GitLab.^33^ Successive versions are archived on Zenodo^15^ under a CC-BY license (unless indicated otherwise) for integration in other resources, such as patRoon^14^ and FAIR-TPs. The terminology used in this article matches the fields from the source data; i.e. parent or precursor compounds are termed “predecessor” and TPs/metabolites are termed “successors”.

The integrated compound-level data stored in the *Transformations_CID_Info_all.csv* file on Zenodo^15^ includes identifiers and structural information such as the PubChem Compound Identifier (CID), InChI,^34^ the hashed InChIKey^34^ and the first block of the InChIKey (IKFB) and the SMILES.^35^ The identifiers are described in more detail elsewhere.^36^ Other associated data includes the molecular formula, exact mass, and the predicted octanol–water partitioning coefficient retrieved from PubChem (XlogP). Finally, the names include the IUPAC name and titles provided by PubChem.

The reaction information in the *PubChem_all_transformations_wExtraInfo.csv* on Zenodo^15^ contains detailed metadata describing the chemical transformations between compounds. This includes two forms of reaction SMILES (ReactionSMILES, DescReactionSMILES), biosystem, enzyme, transformation (a descriptive label), MassDiff and XlogPDiff. The last two aid in compiling statistics. The predecessors and successors are joined to the compound-level data via CID, while the names in the predecessor and successor fields are collected for the consensus name. Source information includes paired fields for each of three reference collections: data set (datasetref, datasetdoi), evidence (evidenceref, evidencedoi), and source (sourcecommentfull, sourcecomment). The data set refers to either ChEMBL or the NORMAN-SLE collection, the evidence is the article where the data was retrieved, whereas the source is the source of the original information. Each contains a text field (first), followed by an identifier field (second). Since source data are not always a publication with a DOI, the latter field was made more flexible to allow for URLs or other identifiers. These headers were decided during the PubChem integration.^11,12^ Citation guidance is included in the frequently asked question (FAQ) section of the website (https://fairtps.lcsb.uni.lu/faq/).

### 2.2 Pipeline Model

FAIR-TPs was designed to make transformation knowledge accessible, testable, and reusable, providing functions to explore and produce outputs suitable for scientific dissemination. The design implementation consisted of two main parts: (1) graph-based storage since the compounds and their reactions are highly interconnected, and (2) display of the graphs through an interactive web interface with statistics and multiple search capabilities. FAIR-TPs is built on a Neo4j (v5.25) graph,^37^ a Django (v5.2.4) web backend,^38^ RDKit (v2025.03.0) for cheminformatics functionality,^39^ and a client-side interface for interactive visualization and downloads. Further details are given in the FAIR-TPs GitLab repository (https://gitlab.com/uniluxembourg/lcsb/eci/fairtps_web).^40^

Figure 1 shows the modular, reproducible pipeline underpinning FAIR-TPs that is rerun as new versions are released on Zenodo. During the Extract step, a Python Programming Language (RRID:SCR_008394) script retrieves the latest version of the source files, which are then cleaned and normalized in a chemistry-aware manner during the Transform step (standardizing identifiers, homogenizing names based on frequency and safely converting data types). In the Load step, records are loaded into the Neo4j database based on the graph model, while constraints/indexes are created to enable efficient paths and improve query performance. The Process step then runs statistics queries to check the consistency and completeness of the data to see if all data are loaded correctly. In the fifth step, the results are presented by a Django-based Web User Interface (UI), which is finally deployed to the public instance (https://fairtps.lcsb.uni.lu/). Each step is explained in more detail below.

**Figure 1:**
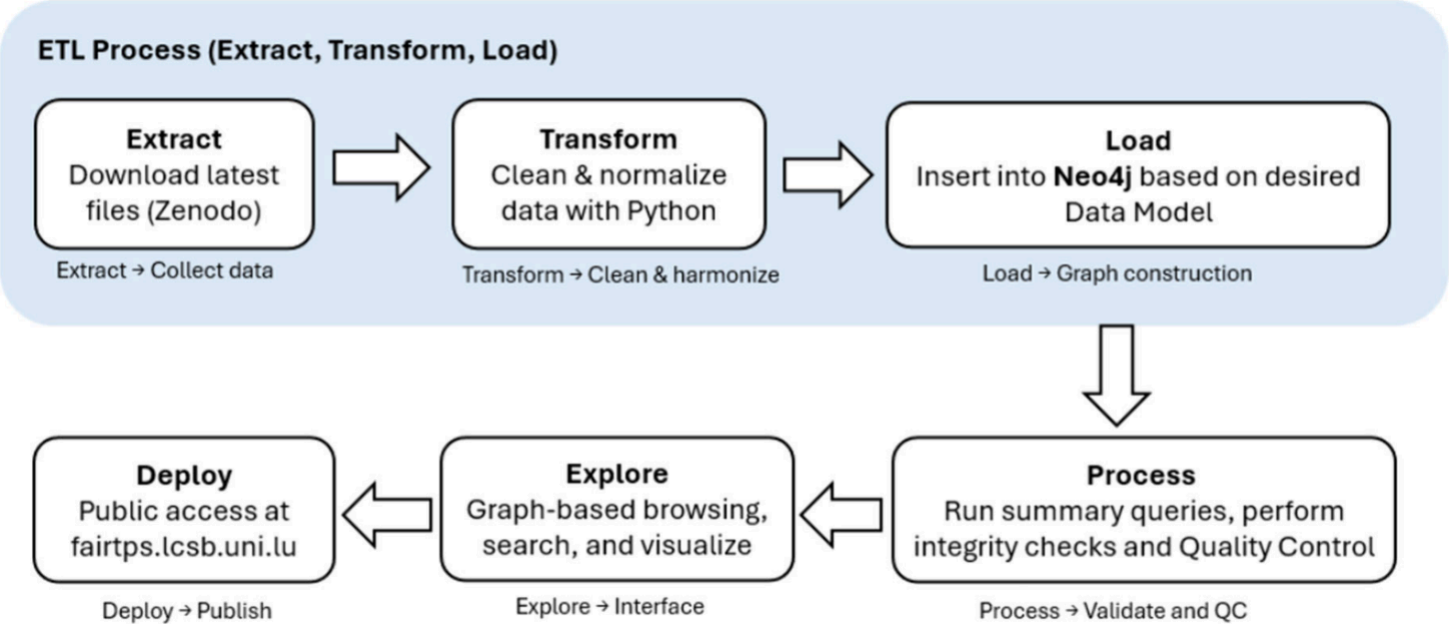
Reproducible FAIR-TPs Pipeline: From data source (Zenodo)^15^ to interactive web interface (https://fairtps.lcsb.uni.lu/).

***Extract:*** The transformation data in FAIR-TPs come from the “Transformations in PubChem - Full Dataset” hosted on Zenodo^15^ under a CC-BY license, created as described in the “Source Data” section. Two main files (both csv format) are used: *Transformations_CID_Info_all.csv*, which contains compound details and *PubChem_all_transformations_wExtraInfo.csv*, which contains reactions details. The current data set used in FAIR-TPs (v 0.2.2) was released on November 22, 2025 and includes 11,190 reactions related to 9,435 distinct compounds.

***Transform:*** In this step, preprocessing steps are applied to prepare the data for loading with chemistry-aware normalization: all identifiers and structures are homogenized, and basic data checks are performed (removing extra spaces, resolving character format issues). Since contributing data sources can use a variety of names, a majority-vote naming approach is used that systematically prioritizes names based on length (preferring shorter, more informative names) and frequency of mentions across different sources and reactions (e.g., 6:2 FTOH is used instead of 3,3,4,4,5,5,6,6,7,7,8,8,8-Tridecafluorooctan-1-ol). This improves clarity across the graph and website, while all names are retained for efficient searching. Very long names (>100 characters) or names containing >20 special characters are trimmed and replaced with the PubChem identifier (format “CID [number]”) for display purposes.

***Load:*** Since transformation data follows a path-like structure (compounds connected via directed reactions), a graph database schema was chosen over a relational database schema to avoid heavy join queries for basic tasks. As the Cypher query language allows efficient navigation across interconnected, complex relationships, Neo4j was chosen to store the data as a network of nodes and edges rather than in tables like a relational database. A minimal, transparent graph is used for better querying. Each *compound* is considered a *node*, while each *reaction* forms a *directed edge* connecting a single predecessor to one successor. Figure 2 shows the example of Acetamiprid, with the compounds as nodes and the reactions as edges. Nodes (with structural and descriptor information) are generated from the *Transformations_CID_Info_all.csv* file, with the majority-vote name used for display. Edges are generated from the reaction data contained within the *PubChem_all_transformations_wExtraInfo.csv* file, with each edge representing a predecessor-to-successor relationship.

**Figure 2:**
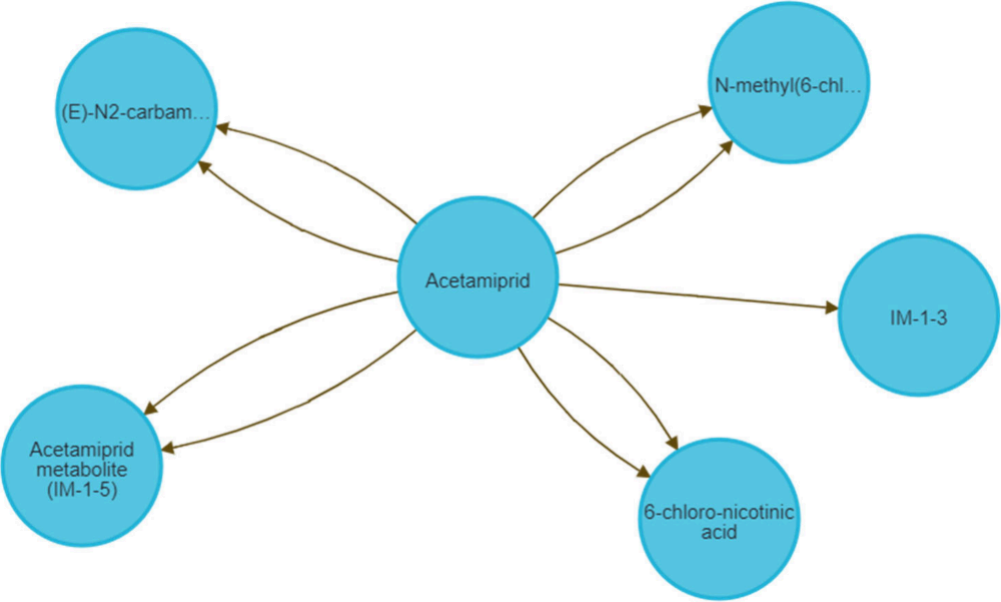
View of compounds directly connected to Acetamiprid with different reactions in Neo4j. Each node represents a compound, and each arrow (edge) represents a reaction stored in the database. Nodes can be connected by more than one reaction if multiple reactions for those compounds are present in the database (e.g., for different biosystems).

The three ***Extract***, ***Transform***, and ***Load*** (ETL) steps (blue shaded in Figure 1) are performed by a Python script , which reads the data in csv files from the Zenodo source, applies the transformations, and finally stores them as a graph in Neo4j.

***Process:*** This stage builds an accurate picture of the graph contents following the ETL phase, then verifies the reliability and reproducibility of the numbers for the statistics page (https://fairtps.lcsb.uni.lu/stats/). The number of nodes and edges recorded in Neo4j is verified with the statistics on Zenodo, while the connectivity of all edges to a valid CID is also checked (no missing end points). Verification points include checking that versions/DOIs match between the deployed instance and Zenodo, as well as key summary statistics such as the total number of compounds and transformations (further details are given in the FAIR-TPs code repository ^40^). In the graph dimension, the degree of input and output of each node (number of reactions per compound), the number of neighbors, the role of nodes (predecessor, successor or both), and the frequency of multistep paths are calculated. This helps to identify data hubs and gaps. Here, “data hubs” refer to compounds that act as highly connected nodes in the transformation graph (i.e., high in-/out-degree), often representing small molecules that can link otherwise weakly related parts of the network. Identifying data hubs helps establish filters for viewing and searching the data, as well as detecting potential artifacts in the data. The calculation of the smallest component, the degree of connectivity of graphs to each other, the average number of reactions per node, etc., is also performed during this step. The results are entered into the statistics page (https://fairtps.lcsb.uni.lu/stats/) and are available for download.

***Explore:*** The core of the web interface is a Django application that communicates with Neo4j and produces HTML page output, graph-specific JSON, and CSV/XLSX files for download as needed. Molecule images are rendered on demand from SMILES as SVG using RDKit; these images are used for thumbnails and chemical structures throughout the website. The detailed compound page contains identifiers, display name, formula, etc. along with a lazy-loaded interactive graph panel with controlled depth and multiple display options to ensure quick loading. High-quality JPG image or data export as CSV, XLSX, patRoon and MetFrag^41^ formats are offered. All variable-length traversals are depth-limited such that the (time-consuming) full subgraph is loaded only when requested by the user. CIDs are considered unique (to ensure that each compound appears only once), while indexes were created on other key identifiers (InChIKey, InChI, Name, and SMILES) to speed up searches in the large graph. All SMARTS^42^ queries (described below under substructure search) are performed on the server side; only the results are sent to the user browser to maintain synchronization of the experience across devices. The final output of the entire pipeline is a fast, resource-based user interface that provides rich information about compounds and their reactions with the ability to view and explore them in an interactive environment with an optimized search for research purposes.

***Deploy:*** The publicly accessible FAIR-TPs web application is hosted on a virtual machine (VM) located at the University of Luxembourg, available at https://fairtps.lcsb.uni.lu/. The VM runs Ubuntu 22.04.5 LTS (x86_64) on VMware with a 4-core Intel Xeon Gold 6246 @ 3.30 GHz processor and 8 GB of RAM. It uses Linux kernel 5.15.0–153-generic, with NGINX (engine-x)^43^ handling caching and static content serving to improve performance. Regular maintenance, releases, and ongoing support ensure website availability.

## 3 Results

### 3.1 FAIR-TPs Web Interface

The FAIR-TPs interface is designed to transform the underlying graph database into a smooth browsing experience to explore TPs and metabolites collected from open and trusted sources in a manner complementary to existing resources. It is based upon three different searches: “ Explore All TPs ”, “ Substructure Search ” and “ Shortest Pathway ”, all accessible from the landing page (see Figure 3). Each search has single/batch search and filtering options, examples to get new users started, and several download options for the results, described further below. The compound page also offers several options to explore the reactions.

**Figure 3:**
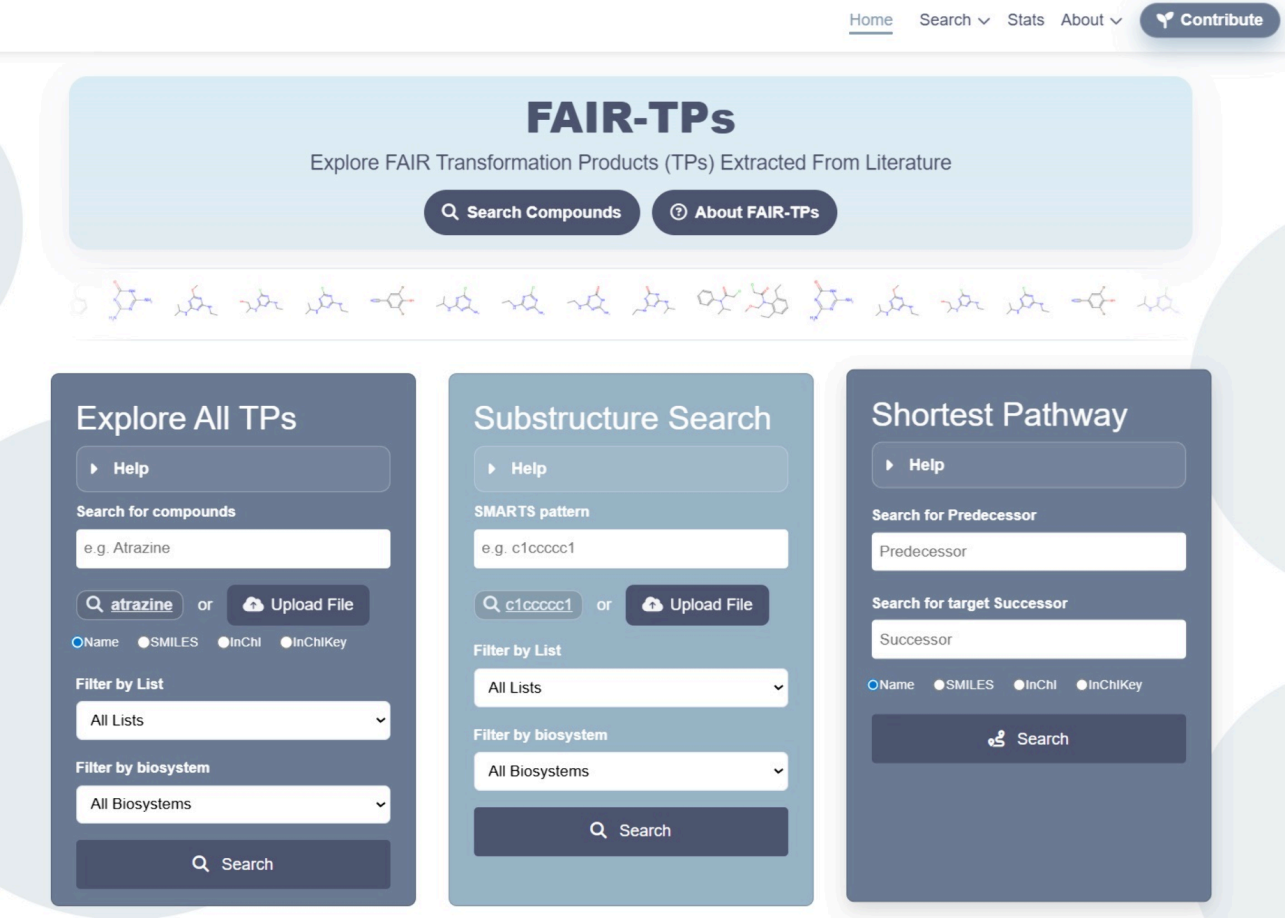
FAIR-TPs landing page, with three basic search functions ( Explore All TPs , Substructure Search and Shortest Pathway ) and menu options to browse statistics , details about the website and how to contribute . Use embedded hyperlinks to land on each page.

***Explore All TPs:*** This search option allows users to search for a single compound directly in the search field (e.g., atrazine ) or a list of compounds by uploading a textfile with compound identifiers. The results show the compound(s) in the search, any predecessors/TPs of that compound, as well as other compounds sharing the same predecessors/TPs. The search results can be filtered by biosystem via a dropdown menu. It is also possible to view the individual predecessors/TPs per compound (see Figure 4, example of TFA ), as well as the details of any of the predecessors/TPs and the reactions from the reaction network. To keep the compound information display compact, users are linked to further information on the external PubChem^10^ and PubChemLite^12,44^ pages by CID.

**Figure 4:**
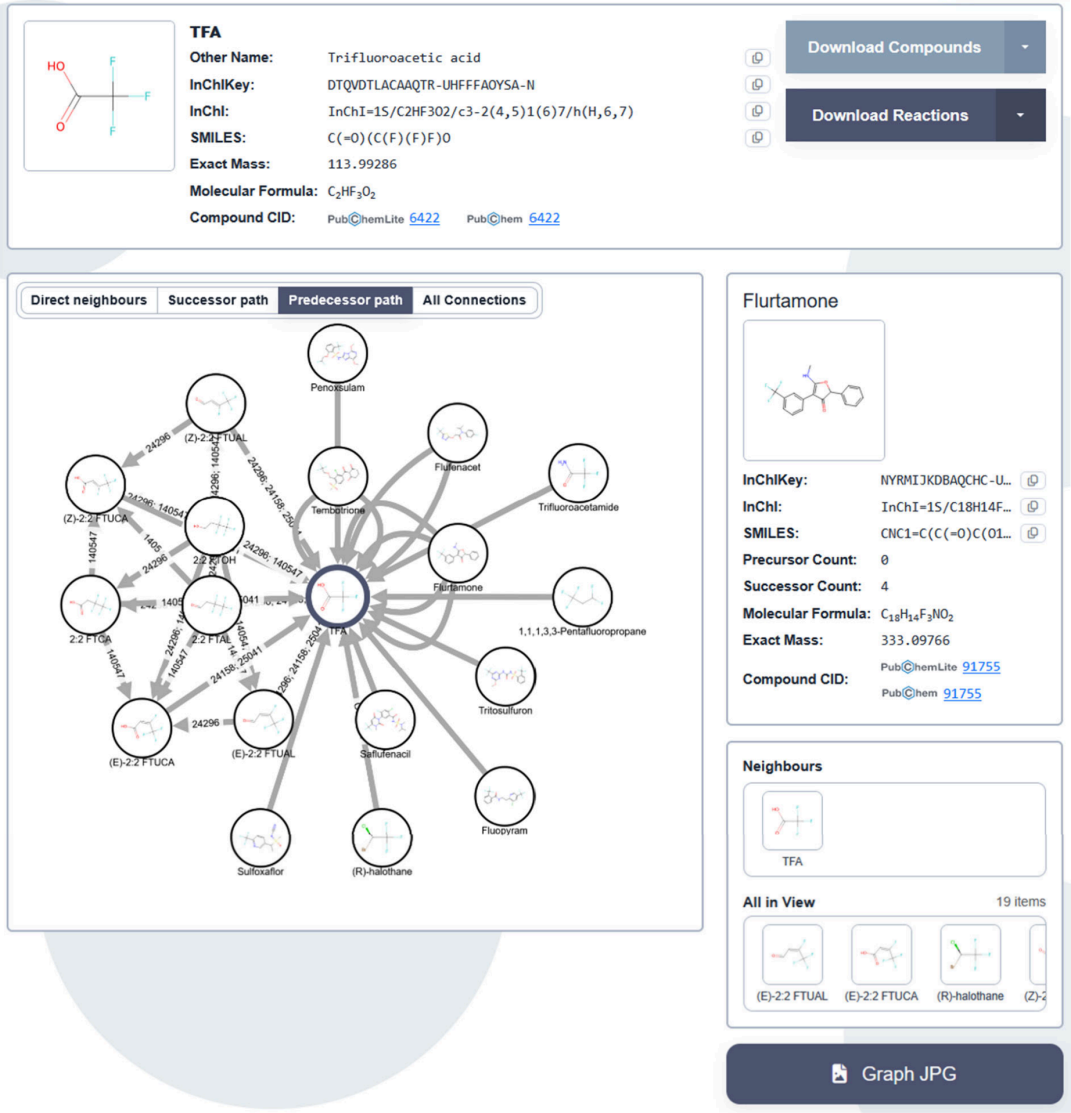
Compound view for TFA , showing the predecessor path view (network), details for the predecessor Flurtamone, and thumbnails for neighbors and all in view (bottom right). Each node represents a compound, while each arrow (edge) represents a reaction stored in the database. Nodes can be connected by more than one reaction if multiple reactions for those compounds are present in the database (e.g., for different biosystems).

***Substructure Search:*** This allows the user to search for compounds with a given substructure (or substructures) using SMARTS,^42^ which is useful for investigating parents and/or TPs belonging to certain compound classes, e.g., any CF _3_ -containing PFAS or triazines . The search results function similarly to the ***Explore All TPs*** search above but also show which substructures are present in the compounds. Most substructure queries return results within milliseconds due to the small database size (9,435 compounds, 11,190 reactions) and directed graph storage in Neo4j, with e.g. even batch runs of 1000 SMARTS queries run on the VM completing in <1.14 s. Any longer perceived delay is typically due to client-side rendering rather than the query itself.

***Shortest Pathway:*** This search returns the reaction pathway with the fewest number of reaction steps between a predecessor and successor using directed reactions (predecessor → successor). All steps are counted equally, with no weighting applied. When there are several equally short pathways, all possible shortest pathways are listed for users to browse (see e.g. dibutyl phthalate → catechol). Autocomplete is used in both fields (predecessor, successor) to show best matches with the data set to reduce ambiguity. Entering a predecessor allows the user to see all possible successors. Once a successor is specified, the shortest path between the two compounds will display (e.g., atrazine → ammelide ). As for the substructure search, the shortest pathway queries typically run within milliseconds due to the small database size.

***Compound view:*** Each compound page has four options for displaying the transformation product networks (predecessor and successor relationships). These are the Direct neighbors, Predecessor path, Successor path and All connections options. The Direct neighbors graph shows the predecessors and successors within a single reaction step as given in the data (note that several “single reaction step” entries recorded in the source data may require multiple transformations). The Predecessor path page shows all of the predecessors (i.e., possible parents) of the compound, including multiple reaction steps. The Successor path shows all successors (TPs) in multiple reaction steps for the compound. The All connections graph shows the full connected component in the database, including all reaction steps of predecessors and successors for that compound as well as other compounds connected to the same successors and predecessors. The page also shows the identifier information (e.g., SMILES and name) of the compounds involved in the reaction when these compounds are selected (see Flutamone in Figure 4). Similarly, the reaction metadata for a given reaction can be displayed by clicking on the arrows/edges. Various download options are available, as described above.

### 3.2 Data Overview and Statistics

The statistics page of FAIR-TPs (https://fairtps.lcsb.uni.lu/stats/) gives a live overview of the data. Of the 9,435 compounds in the FAIR-TPs database, 1,981 compounds are predecessors (parents), 5,624 are successors (TPs) and 1,830 are both. The number of reactions associated with these compounds varies greatly: 122 compounds have 10 or more direct TPs, while 2022 compounds only have one known documented TP. Similarly, only 26 compounds have more than 10 known direct predecessors, while 6,519 compounds have only a single documented direct predecessor. The compound with the most known direct TPs is ranolazine (39) followed by nicotine (34) and bupropion (27), while carbon dioxide (36), formaldehyde (29) and PFOA (24) have the most known direct predecessors (see Figure 5A). PFOA likely appears prominently due to a concerted effort to capture PFAS reactions in REFTPS (including high energy treatment options, which are part of the data set due to water treatment being a source of water contamination). Several predecessor-successor pairs are represented in multiple data sets. In some cases, these are multiple reports of the same reactions from the same source and reference (e.g., several diclofenac entries in ChEMBL), while in other cases these “duplicates” show reactions in different biosystems, with different enzymes or from different references. Duplicate reactions are indicated with multiple arrows in the compound view (see Figure 4, right of center). The reaction metadata can be viewed by clicking on the respective arrows to investigate the differences in the reactions. Although duplicates are not currently filtered in FAIR-TPs, this is a potential future development, especially as the data set grows in the future.

**Figure 5:**
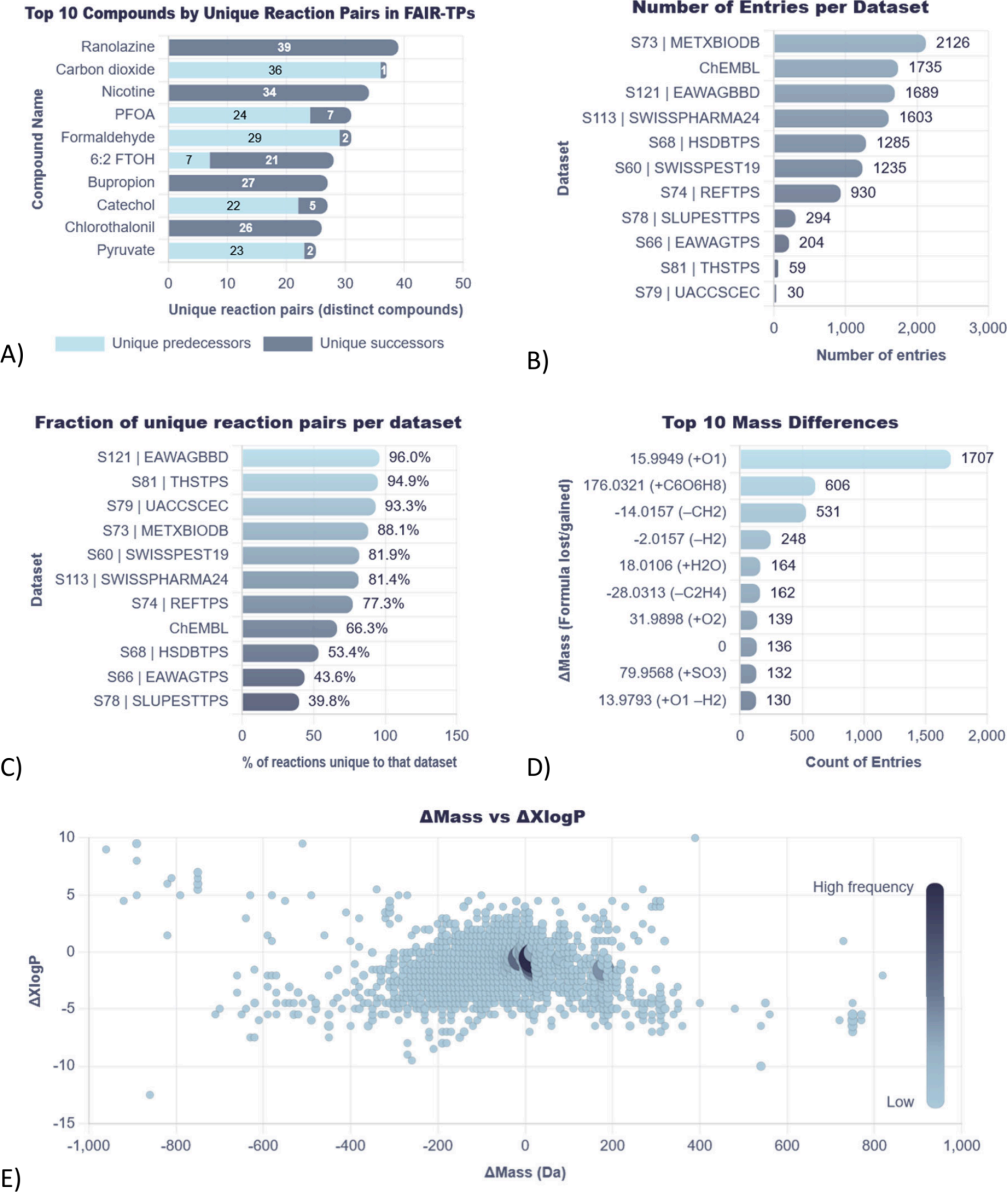
FAIR-TPs statistics. (A) Top 10 compounds by unique reaction pairs. (B) Number of entries per data set. (C) Fraction of unique reaction pairs per data set. (D) Top 10 mass differences. (E) Distribution of the change in mass vs change in XlogP.

The statistics page also provides an overview of each data set (see Figure 5B). While the largest data sets (METXBIODB, ChEMBL, EAWAGBBD and SWISSPHARMA24) contain the highest number of unique reaction pairs, some of the smaller data sets have a larger fraction of unique reactions relative to the total number of reactions in those data sets (see Figure 5C). For example, 95% of the reactions found in THSTPS (specifically focusing on thirdhand smoke metabolites) and 93% of the reactions in UACCSCEC (several emerging contaminants such as plasticizers and flame retardants) are not found in any of the other data sets, showing the value of small, curated data sets in expanding the coverage of FAIR-TPs.

There are several ways to view the most common types of transformation reactions, with the example of mass difference shown in Figure 5D. Mass differences can help connect unidentified masses to known parents/TPs in nontarget data, but also reveal the potential reaction type. Oxygen addition is the most common reaction, followed by glucuronidation and demethylation. More details are given on the statistics page. Figure 5E shows the distribution of mass difference versus difference in predicted XlogP, another distribution very interesting for NTS experiments, as this provides information on both mass and retention time (which correlates with XlogP in reversed phase chromatography). Most TPs have a lower XlogP, which matches observations in the literature.^45^ Figure 5E also indicates some TPs with much lower and much higher mass compared to their predecessors. These are not artifacts, but a reflection of the source data: most of the reactions with >600 Da difference are related to degradation of coenzymes to small molecules or polymers to their respective monomers, or synthesis of larger coenzymes from smaller molecules.

Some of the data sets contain biological reactions where small compounds like carbon dioxide and acetate are involved in the formation of much larger compounds. While these reactions are correctly reported in the data, they also present a challenge for the shortest pathway searches and the compound pages. In the shortest pathway search, this results in some reaction pathways that do not normally occur in the environment such as the formation of “2-acetamido-2’-benzoyl-4’-chloro-*N*-methyl acetanilide” from bisphenol A. This same problem exists in the “all connections” display, where two otherwise unrelated data islands are connected, making the graph more complicated to read. To alleviate these issues, a filter was implemented to prevent the display of reactions where a compound with a mass of less than 60 Da is the precursor of a compound with a mass of more than 300 Da. The lower threshold of 60 Da was chosen to exclude molecules such as CO_2_ and acetate, but retain environmentally relevant molecules such as triazole (69 Da). The upper threshold was selected on the basis that this value excluded all of the problematic reactions, without excluding other transformations. For the current version of the data set, this results in 6 reactions that are “hidden” from the displays on the compound pages and the shortest pathway search. These 6 excluded reactions are listed on the stats page (bottom right panel) and are Acetate → N-(2-benzoyl-4-chlorophenyl)-2-acetamidoacetamide; Acetate → N-(2-benzoyl-4-chlorophenyl)-2-acetamido-n-methylacetamide; Carbon dioxide → Formylmethanofuran; Acrolein → ChEMBL3706542; Ethylene oxide → Acetyl-CoA; Formaldehyde → S-hydroxymethylglutathione. No modifications have been made in the source data, such that these reactions can still be found if they are of interest to the user.

## 4 Discussion

The FAIR-TPs website provides both a detailed display of multistep transformation reaction pathways as well as an overview of the type of transformation reaction data available in public data sets. Obtaining this information overview is important for identifying the available information and gaps in the TP data. Looking in more detail at the contributing data sets yields some insights into the type of compounds in the FAIR-TPs database. Some of the largest data sets are transformation reactions involving pharmaceuticals such as SWISSPHARMA, ChEMBL and MetXBioDB, the data set behind BioTransformer. SWISSPEST and SLUPESTTPS focus exclusively on pesticides, whereas HSDBTPS and EAWAGTPS are a mix of pesticides, pharmaceuticals, and other contaminants relevant for toxicological and/or environmental studies. The REFTPS data set has been used to fill gaps in open TP data, with a strong (but not exclusive) focus on PFAS, enhanced by other recent literature contributions, whereas THSTPS focuses exclusively on thirdhand smoke metabolites, and UACCSCEC contains data on plasticizers and flame retardants.

Overlapping the 1,981 predecessor compounds in FAIR-TPs with various categories from the PubChem classification browser (https://pubchem.ncbi.nlm.nih.gov/classification/#hid=72) confirms this distribution. In total, 816 (41%) are categorized as pharmaceuticals/drugs, 414 (21%) as pesticides, 160 (8%) PFAS, 128 (6%) lipids, and 110 (5.5%) cosmetics. Note that some categories overlap (e.g., some pharmaceuticals are also classified as pesticides and can also be used in cosmetics for antimicrobial properties). Other compound classes such as industrial compounds and air pollutants seem underrepresented in comparison, which shows a need for further contributions of open data and/or additional research into the environmental and metabolic fate of these compounds. Although more data exist in FAIR-TPs on pharmaceutical transformations than pesticides, the relative proportion of information for these chemical classes in PubChem is quite different. Of the 3,022 agrochemicals (pesticides and TPs) in PubChem, 1,681 (56%) have transformation information available, whereas only 1,364 of 17,920 (7.6%) pharmaceutical entries have transformation information available. For PFAS this proportion is much worse, with information available for only 694 of the 7,299,515 PFAS in PubChem (0.0095%).

One of the main limitations of some sources of information on TPs (e.g., PubChem Transformations and CTS) is that they only show a single reaction step, while others that show multiple reaction steps are limited to a single database (e.g., ChEMBL and enviPath) FAIR-TPs solves this problem by showing multistep transformation pathways from 11 publicly available transformation data sets. This allows viewing of longer transformation reaction pathways. The data set contains 1,997 compounds that are 2 or more reaction steps away from any “original predecessor” compound (a compound that has no predecessors). For 57% of these compounds, 2 or more data sets are included in the reaction pathways. This shows that the ability to display multiple reaction steps from multiple references is crucial to obtain a complete picture of the TPs and/or predecessors of any given compound to track their environmental and/or metabolic fate. Being able to visualize multiple reaction steps from multiple data sets also facilitates discovering potential sources of different TPs. For example, 7 out of 9 pesticide predecessors for cyanuric acid (a compound of concern due to persistent, mobile, and toxic (PMT) properties) are 2 or more reaction steps away. These would easily be overlooked in sources only displaying a single reaction step but are easily visible in FAIR-TPs. The reaction pathways of cyanuric acid involve 7 out of the 11 data sets, which again shows the importance of forming a combined database from multiple data sets.

In the context of nontarget and suspect screening, linking TPs formed with multiple reaction steps to their precursors is possible with the patRoon TP workflow, but only if the predecessors are detected. Thus, if the original predecessor compounds are not found, the connection could easily be missed. For these compounds, FAIR-TPs could be used to identify potential parent compounds that for various reasons (e.g., complete degradation) were not detected in the samples. The download files provided by FAIR-TPs are directly compatible with the nontarget and suspect screening workflows in patRoon and MetFrag for easy integration into these workflows. The MetFrag export contains Identifier, Name, InChIKey, InChI, SMILES, MolecularFormula, and MonoisotopicMass columns, while the patRoon export contains Name, InChI, SMILES, Formula, and ExactMass columns.

FAIR-TPs could also be used to inform the TP prediction models. This includes both knowledge based models such as BioTransformer^16^ and enviPath^6^ as well as machine learning (ML) models (although both BioTransformer and enviPath also contain ML components). Knowledge based models rely on experimental data for the development of reaction rules that the model applies to the input structures. These rules are commonly defined as SMIRKS^46^ patterns, where the program searches for the given substructure in the predecessor compound and, when present, applies the changes shown in the successor substructure. For this purpose, FAIR-TPs may be particularly effective in helping define new substructure reaction rule patterns. ML-based models, such as those for organic synthesis, commonly use neural network algorithms, which require large training data sets.^47^ While the data in FAIR-TPs are likely insufficient for such models, the database does provide an open collection of literature data for transformation reactions that can be expanded through data sets submitted by users. This is essential to increase the available TP data needed for developing ML models.

### 4.1 Contributing Reactions to FAIR-TPs

As seen from the fraction of unique reactions in the data sets, even smaller transformation data sets can fill important data gaps. Still much TP data remains underutilized because it is not made easily available in a FAIR format. To make transformation reaction data FAIR, it is essential that it is reported with structural information such as SMILES (preferred) and/or InChI together with the reaction conditions (if they are known) and made available in a file format that is easily machine readable such as a CSV file, as discussed elsewhere.^36^ The proposed template by Schymanski and Bolton^36^ is the one used for uploading data to PubChem and the default suggested template for submission to FAIR-TPs. Another more extensive template for reporting of transformation reactions is the BART template^48^ used for uploading transformation reactions to the enviPath data set. Both templates describe the predecessor/successor connectivity using their names and SMILES, and allow for some reporting of the conditions under which the reaction takes place. For the FAIR-TPs template this is done in the biosystem field, while for the BART template separate Excel sheets with 35 to 102 fillable parameters are designated for sludge, soil, water-sediment, and general conditions. Thus the BART template is more specialized for soil and water sampling with significantly more meta data. While this information is highly valuable, for example, in machine learning applications, the template is not easily generalizable to other sample types and is more time-consuming to complete. The FAIR-TPs template, on the other hand, is applicable to any sample type and requires less meta data, which facilitates easier reporting of TP data for purposes such as nontarget and suspect screening, but provides less rich information. Both templates can be used to provide data to FAIR-TPs, as described on the website (https://fairtps.lcsb.uni.lu/contribute/).

## 5 Conclusion

FAIR-TPs was designed to make TP data from multiple data sets easier to use and explore. It allows for visualization of full reaction pathways, which makes it possible to trace the fate of different contaminants and potentially group predecessors of hazardous TPs. It is possible to download all reactions in formats compatible with patRoon and MetFrag for easy integration into nontarget and suspect screening workflows. The statistics page provides an overview of the types of compounds and reactions in the data set, which can help identify research gaps.

The database is set up to easily include new data from contributors, and submission of new data sets via the templates linked on the website is encouraged. As discussed above, even smaller TP data sets can help fill significant knowledge gaps. Feedback is welcome and will help prioritize additional features for future developments.

## Data Availability

The data underlying this study are openly available in Zenodo at DOI 10.5281/zenodo.17682349. These data were derived from sources in the public domain as described in the Methods section. The website is available at https://fairtps.lcsb.uni.lu/. The code is available at https://gitlab.com/uniluxembourg/lcsb/eci/fairtps_web.
